# An Incidental Necropsy Finding: Intrathoracic Ectopic Liver in a Cat

**DOI:** 10.3390/ani16050742

**Published:** 2026-02-27

**Authors:** Joanna Fiedorowicz, Katarzyna Paździor-Czapula, Mateusz Mikiewicz, Iwona Otrocka-Domagała

**Affiliations:** Department of Pathological Anatomy, Faculty of Veterinary Medicine, University of Warmia and Mazury in Olsztyn, Oczapowskiego 13, 10-719 Olsztyn, Poland; katarzyna.pazdzior@uwm.edu.pl (K.P.-C.); mateusz.mikiewicz@uwm.edu.pl (M.M.); i.otrocka-domagala@uwm.edu.pl (I.O.-D.)

**Keywords:** ectopic liver, cat, hepatic choristoma

## Abstract

Ectopic liver is a rare anomaly characterized by the presence of hepatic tissue outside its normal anatomical location without continuity with the main liver. Reports of this condition are sporadic in human medicine, and cases in veterinary species remain limited. This condition may arise as a congenital developmental defect or as a consequence of prior traumatic injury leading to fragmentation and displacement of hepatic tissue. This case study describes an incidental intrathoracic ectopic liver identified in a 6-year-old male cat that died of acute suppurative bacterial pneumonia. Necropsy revealed a well-demarcated, encapsulated, large mass within the middle of the caudal mediastinum, unattached to the diaphragm, lungs, or pericardium. Cytologic and histopathologic examinations confirmed the presence of hepatic tissue with preserved lobular architecture. No evidence of previous trauma or diaphragmatic defects was present, supporting the congenital origin. The accompanying severe bacterial pneumonia was unrelated to the mediastinal mass. This study highlights the diagnostic challenges associated with intrathoracic ectopic liver, which can mimic neoplastic or inflammatory masses. Awareness of this entity is essential for accurate diagnosis. Given the documented risk of malignant transformation in ectopic hepatic tissue, surgical excision and histopathological assessment should be considered whenever such lesions are detected.

## Simple Summary

The presence of liver tissue outside its normal anatomical location is defined as ectopic liver. In human medicine, it is an infrequent abnormality, and only a few cases have been reported in veterinary medicine. Ectopic liver is usually asymptomatic; however, previous studies have observed a higher risk of carcinogenesis in individuals with this condition, especially the development of hepatic carcinoma. This case study presents an incidental post-mortem finding of a large (9 cm) intrathoracic mass in a 6-year-old cat that died suddenly due to severe bacterial pneumonia. Cytologic and histopathologic examinations of the mass confirmed the presence of ectopic liver tissue. We briefly discuss the main findings in veterinary medicine regarding ectopic liver, including anatomical localization, reported species, mass sizes, histopathological features, and associated clinical symptoms. Although ectopic liver is rare, it can be easily misdiagnosed as a tumor mass, which may influence further clinical decisions. Therefore, it is important to increase awareness about this entity and improve clinical management.

## 1. Introduction

An ectopic liver, or hepatic choristoma, is hepatic tissue located outside the normal anatomic location of the liver [1]. This entity is rarely diagnosed in humans, mostly within the gallbladder wall [2]; similarly, only a few cases have been reported in the veterinary literature, mostly in the thoracic cavity [3,4,5,6,7,8,9,10,11].

Ectopic liver can represent a congenital anomaly or result from trauma, leading to the defragmentation and displacement of hepatic tissue [2]. Typically, it is asymptomatic and found incidentally during necropsy or diagnostic imaging. However, the possible predisposition of ectopic liver to hepatocellular carcinoma has been reported [12,13,14]. Ectopic liver may pose a diagnostic challenge, especially in diagnostic imaging, where it can be easily misdiagnosed as a tumor [8]. This report presents an unusual case of probably congenital ectopic liver in a cat, manifested as a large, intrathoracic tumor-like mass, found incidentally during necropsy.

## 2. Case Description

A 6-year-old male, obese European Shorthair cat, was presented to a veterinary clinic with hypersalivation caused by a tooth abscess and severe dental calculus. This was accompanied by mild enlargement of the submandibular lymph nodes. The cat was administered with cefovecin (8 mg/kg s.c.) at the visit, and meloxicam (0.1 mg/kg p.o./every 24 h) for the next 5 days, and a follow-up visit was scheduled for 5 days. Unexpectedly, the cat died suddenly 3 days later without any apparent clinical signs, except for mild lethargy directly before death, and was submitted for necropsy.

The last routine blood test was performed approximately nine months before death, which revealed nonspecific, minimal hematological and biochemical alterations. In hematology, a slightly elevated mean corpuscular volume (MCV) 60.4 fl (38.0–54.0 fl) and mildly elevated mean corpuscular hemoglobin (MCH) 18.2 pg (11.8–18.0 pg) were recorded. In biochemistry, similar situations were observed with subtle increases in glucose to 131 mg/dL (100–130 mg/dL) and total proteins (TP) 8.3 g/dL (6.0–8.0 g/dL). This is most likely related to procedural stress and transient dehydration. Hepatic enzymes, such as alanine aminotransferase (GPT), aspartate aminotransferase (AST), and alkaline phosphatase (ALP), were within their reference ranges. According to the owner, no health problems were detected during the cat’s lifetime. The cat was routinely vaccinated and underwent a clinical examination.

**Figure 1:**
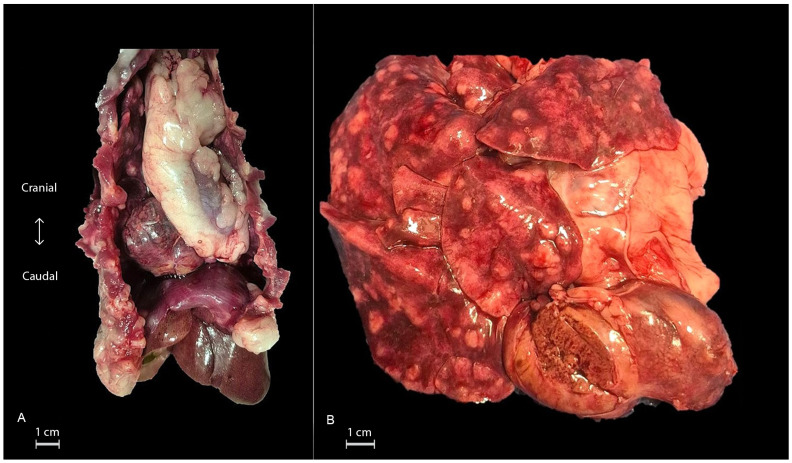
(**A**) Photograph of the open thoracic cavity with an intact diaphragm and liver. The mass is attached to the sternal adipose tissue and occupies the middle and caudal mediastinum. (**B**) Photograph of the isolated lungs with surrounding adipose tissue and the mass. In the lungs, there are multifocal to coalescing white nodules, observed in all lobes. On cross-section, the mass resembles mild congested liver tissue.

Gross examination revealed a firm, encapsulated, brown oval mass measuring 9 × 4 × 3 cm, localized in the middle/caudal mediastinum, attached to sternal fat tissue, without any macroscopically visible connection to the lungs, diaphragm, or pericardial sac. Furthermore, the lungs were affected diffusely by multifocal-to-coalescing, elevated white nodules, measuring up to 2 cm in diameter. Multiple areas of hemorrhage were also identified (Figure 1A,B). On the cut surface, the nodules contained white, dense, purulent exudate. The other organs showed no significant lesions. The mass and lungs were sampled for cytology (imprints) and histopathology.

**Figure 2:**
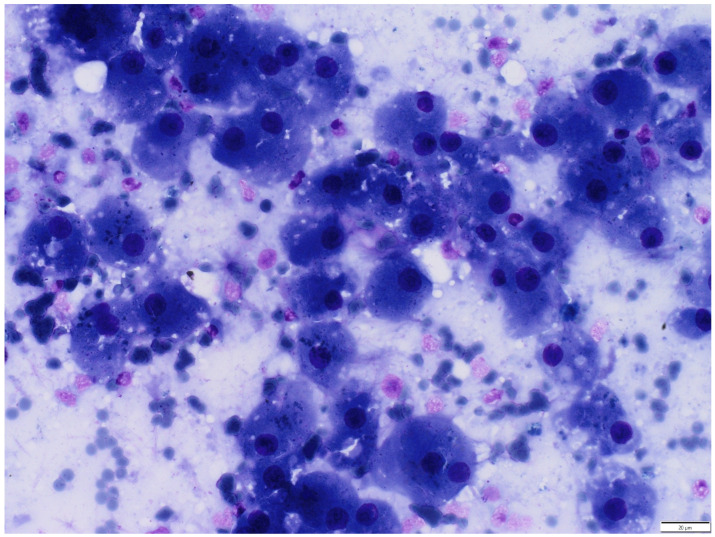
Photomicrograph of an imprint cytology from the mass. There is a high number of hepatocytes, usually containing intracytoplasmic black pigment (most likely lipofuscin) and occasionally clear vacuoles. Binucleation is observed. Hepatocytes are accompanied by a moderate number of Kupffer cells and lymphocytes. In the background, numerous erythrocytes, cellular debris, and proteinaceous fluid are present. Hemacolor ^®^ (Merck KGaA, Darmstadt, Germany).

Cytologic examination of the thoracic mass revealed clusters of polygonal cells with finely granular, light navy-blue cytoplasm and round, dark-purple nuclei with irregular chromatin and occasionally indistinct borders consistent with hepatocytes. The hepatocytes often contained intracytoplasmic black pigments (most likely lipofuscin) and a small amount of clear, minute vacuoles (indicating hydropic degeneration). Additionally, they were admixed with a moderate number of Kupffer cells, small lymphocytes, and neutrophils. In the background, numerous erythrocytes and cellular debris were observed (Figure 2).

Cytologic examination of the lungs revealed a large number of degenerated neutrophils, accompanied by fewer foamy macrophages and small lymphocytes. Both neutrophils and macrophages contained phagocytosed bacteria (cocci). There was an abundance of proteinaceous fluids with numerous bacteria and a few erythrocytes in the background.

Samples for histopathology were immediately fixed in 10% buffered formalin, processed routinely using the paraffin method, and stained with Mayer’s haematoxylin and eosin. Additionally, immunohistochemistry was performed on a sample from the mass. This was conducted using Pan Keratin (monoclonal mouse anti-human, clone AE1/AE3/PCK26, ready-to-use, Roche Diagnostics, Mannheim, Germany) as the primary antibody, and 3,3′-diaminobenzidine (ImmPACT DAB Substrate Kit; Vector Laboratories, Burlingame, CA, USA) as the substrate.

**Figure 3:**
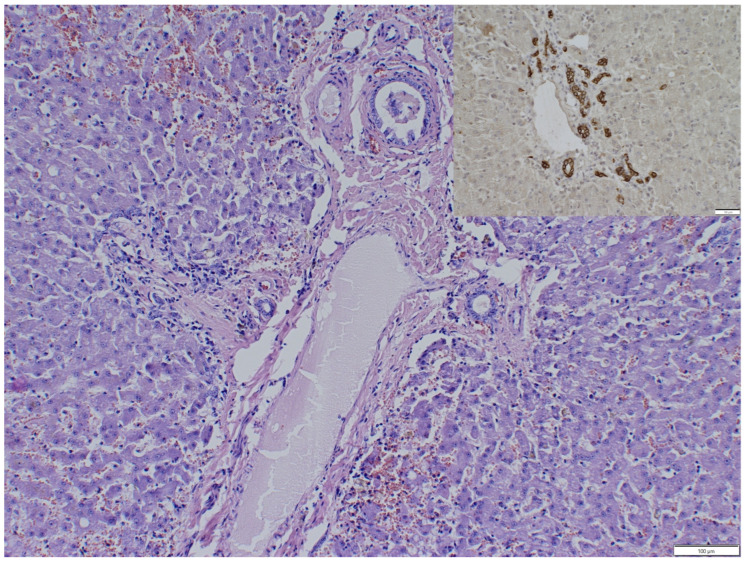
Photomicrograph of a section of the mass. The liver architecture is preserved, with a portal triad surrounded by minimal fibrosis with mild infiltration by lymphocytes, plasma cells, and erythrocytes. Hepatocytes are moderately swollen with abundant eosinophilic cytoplasm, occasionally vacuolated. H&E. Within the portal triad, an increased number of bile ducts are present, which show positive immunoreactivity for pancytokeratin (insert).

Histopathology of the thoracic mass revealed liver tissue with preserved lobular architecture, mild bile ducts, and arteriolar hyperplasia. Hepatocytes of all zones showed acute cell swelling and occasionally vacuolar degeneration, especially in the periportal zones. The sinusoids were moderately congested. Mild multifocal infiltrations of plasma cells and lymphocytes were observed within the hepatic parenchyma (Figure 3).

Histopathology of the lungs revealed multifocal to coalescing, massive infiltrations of degenerated neutrophils; a lower number of macrophages, lymphocytes, and plasma cells accompanied by hemorrhages and compensatory emphysema within the parenchyma. Aggregates of inflammatory cells were observed close to the airways. The lumen of bronchi and bronchioles contained homogeneous, lightly eosinophilic fluid admixed with fragments of exfoliated epithelium with degenerated neutrophils. The alveoli were filled with homogenous, lightly eosinophilic fluid admixed with a reticular meshwork of finely beaded fibrils (fibrin) consistent with alveolar edema. Occasionally, neutrophils and macrophages were identified to contain phagocytosed bacteria (cocci). Additionally, the blood vessels were congested.

Cytology and histopathology on the thoracic mass diagnosed an intrathoracic ectopic liver (hepatic choristoma) with mild bile duct and arteriolar hyperplasia, hepatocellular vacuolar degeneration, and multifocal, mild lymphoplasmacytic inflammation.

Neither bacterial culture nor polymerase chain reaction (PCR) was performed on the lungs, as the owner was not interested in additional examinations. This limited our ability to determine which type of bacteria caused the pneumonia. However, histopathological examination of the lungs revealed the infiltration of multifocal to coalescing inflammatory cells, consistent with suppurative (purulent) multifocal bronchopneumonia, most likely caused by aspiration.

## 3. Discussion

According to the literature, four main types of ectopic liver have been distinguished. The first and second types represent accessory liver lobes, attached to the liver by a stalk, with type I comprising a larger amount of tissue and type II being significantly smaller in size. Type III is a true ectopic liver, without any connection to the anatomical liver. Finally, Type IV describes microscopic ectopic liver tissue, occasionally found in the wall of the gallbladder [1].

The other proposed classification identifies three types of ectopic liver, based on imaging characteristics, which is more adequate for intrathoracic cases. Type I is defined as an extension of the liver tissue through a diaphragmatic defect. In Type II, an accessory liver is connected to the anatomic liver by a pedicle that has vascular and biliary components. In Type III, the ectopic liver is completely isolated from the anatomical liver, with an independent vascular and biliary system [15]. However, in this classification, based on the definition, Types I and II describe accessory liver, whereas Type III refers strictly to ectopic liver.

Based on the absence of continuity with the orthotopic liver and the lack of a diaphragmatic defect, the lesion in this case study is most consistent with a ‘true’ ectopic liver (Type III in commonly used human classifications). However, because no imaging was performed, classification based on imaging characteristics cannot be confirmed.

In veterinary medicine, no veterinary-specific classification system currently exists for ectopic livers. The number of reported cases is very limited; therefore, human classifications are generally applied.

Theories regarding the presence of hepatic tissue in an ectopic location mainly include the development of abnormalities, such as migration or displacement of precursor hepatocytes during embryogenesis or their entrapment in the foregut region after closure of the diaphragm or umbilical ring [2]. When a hepatic choristoma is located in the thoracic cavity, proposed explanations include consequences of diaphragm herniation [15]. As in the present case, there was no evidence of prior trauma; the diaphragm was anatomically intact, and the proper liver was fully developed (all lobes were complete and intact). As such, the traumatic genesis of this lesion seems unlikely. Indeed, old diaphragmatic traumas can be grossly unnoticeable in some cases, which limits their classification. However, as the owner excluded previous injuries, we can assume that the presented hepatic choristoma most probably represents a congenital developmental anomaly.

The ectopic liver is an extremely rare phenomenon in both humans and animals [1,16]. In the veterinary literature, only a few cases of ectopic liver have been reported, including various anatomical localizations in dogs, cats, cows, and guinea pigs. The highest number of cases has been reported in dogs, with the thoracic cavity being the most common location [6,7,8,10]. A single case was described in the subcutis of the costal region [17] and two cases of ectopic hepatocellular carcinoma were confirmed [12,14]. In cats, all previously described cases of ectopic liver are concerned with the thoracic cavity. In two cases, the ectopic liver was connected with the pericardial sac [4,9]; in one case, it adhered to the epicardium of the right ventricle [11], and in one case, it was attached to the diaphragm [3]. In only one of these cases did the ectopic liver likely result from trauma, diaphragmatic herniation, or subsequent fragmentation and displacement of hepatic tissue [3]. In cattle, ectopic liver has been reported in the umbilical region [18] and within the thoracic cavity [5]. Additionally, one case has been documented in a guinea pig, located within the wall of the gallbladder [16]. In humans, unlike animals, the most common location of ectopic liver is the gallbladder wall, and intrathoracic occurrence is sporadic [2].

The size and shape of the ectopic liver in humans vary moderately, ranging from a few millimeters, sometimes barely detectable macroscopically, to several centimeters, with an average reported size of 17.8 mm [2]. In comparison, reported ectopic livers in veterinary medicine are larger, ranging from a few millimeters in a guinea pig [16] to 6 cm in a cow [5] or 7 cm in a dog [7] and a cat [9]. In the present study, the ectopic liver was massive, a tumor-like mass approaching 9 cm.

In most cases reported in humans, the histological architecture of the ectopic liver resembled that of normal hepatic tissue with a preserved, characteristic hexagonal lobular pattern [2]. However, occasionally, disruption and loss of normal functional hepatic architecture were observed [2]. Comparable findings have been described in animals, with mostly preserved liver architecture, complete portal areas, and centrally located central veins [3,4,5,11] rather than loss of central veins [16] or abnormal architecture [18].

Among commonly reported hepatic histopathological changes, vacuolar degeneration, cholestasis, fibrosis, and hemorrhage were most frequently detected [4,8,10,11]. Additionally, in the present case, slight arteriolar and ductular proliferation was observed, which most likely resulted from the ectopic position and supposed circulatory disturbances. To visualize even small ductular proliferation, differentiate them from small arteries, and check distribution, pancytokeratin immunohistochemistry was performed, as it is a marker of bile duct epithelium. Positive expression was observed predominantly within and around portal areas, with just a few randomly distributed bile ducts in lobules, confirming a true ductular reaction.

An intrathoracic ectopic liver may easily mimic a tumor mass or even a diaphragmatic herniation on radiographic evaluation [8]. In companion animals, mediastinal masses are considered a common pathological finding, with mediastinal lymphoma and thymoma most frequently reported in cats [19]. Among non-neoplastic changes, sternal lymphadenopathy secondary to feline infectious peritonitis should be considered an important differential diagnosis [20].

Previous studies have demonstrated that cytology from this region is a valuable diagnostic tool, with high agreement compared with histopathology [19]. Importantly, in our study, cytology (even when collected post-mortem) clearly shows the presence of hepatocytes, suggesting an important and useful role for this technique in the diagnosis of ectopic liver.

Imaging modalities such as color Doppler ultrasonography, computer tomography (CT), or magnetic resonance imaging (MRI) are other valuable diagnostic methods of thoracic masses. Additionally, if an ectopic liver is suspected, similar parenchymal densities may be identified to those observed in orthotopic liver tissue. Furthermore, imaging techniques such as CT or MR angiography can visualize the venous and arterial blood supply, which is particularly useful for planning surgical procedures [15].

In most of the reported cases, the ectopic liver was found incidentally and did not cause any clinical symptoms or have a negative impact on human or animal health [1,2,16]. Clinical problems were reported sporadically, usually due to compression of the surrounding tissues, torsion, or bleeding [1,2,7]. Coughing, exercise intolerance, and anorexia were the most frequently observed clinical signs in animals [3,6,7].

Interestingly, it has been suggested that the ectopic liver may predispose to hepatocellular carcinoma development. The reasons behind this hypothesis include functional impairments of hepatocytes, such as limited or abnormal blood supply and invalid biliary drainage systems. Therefore, ectopic liver tissue is more prone to chronic congestion, oxidative stress, and metabolic complications, which may provide a basis for carcinogenesis [12,13].

## 4. Conclusions

In conclusion, ectopic liver is a rare finding in veterinary medicine. It may present either as a result of a congenital developmental anomaly or, less commonly, as a result of previous traumatic events. In the present case, the ectopic hepatic tissue was located within the thoracic cavity without any evidence of diaphragmatic injury or prior trauma, suggesting a congenital origin. Although typically an incidental and asymptomatic finding, ectopic liver can pose diagnostic challenges, as it may resemble neoplastic lesions. Therefore, awareness of this entity is important to avoid misdiagnosis. Moreover, given its potential for neoplastic transformation, surgical excision and histopathological evaluation are recommended whenever it is detected.

## Data Availability

The original contributions presented in this study are included in the article. Further inquiries can be directed to the corresponding author.
